# The anti-cancer activity of the mTORC1/2 dual inhibitor XL388 in preclinical osteosarcoma models

**DOI:** 10.18632/oncotarget.10389

**Published:** 2016-07-02

**Authors:** Yun-Rong Zhu, Xiao-zhong Zhou, Lun-qing Zhu, Chen Yao, Jian-Feng Fang, Feng Zhou, Xiong-Wei Deng, Yun-Qing Zhang

**Affiliations:** ^1^ Department of Orthopedics, The Affiliated Jiangyin Hospital of Medical College of Southeast University, Jiangyin City, 215600, China; ^2^ The Department of Orthopedics, The Second Affiliated Hospital of Soochow University, Suzhou 215000, China; ^3^ The Center of Diagnosis and Treatment for Children's Bone Diseases, The Children's Hospital Affiliated to Soochow University, Suzhou, Jiangsu, 215000, China; ^4^ Joint group of Orthopedic Department, Affiliated Hospital of Nanjing University of TCM, Nanjing 210029, China

**Keywords:** osteosarcoma (OS), mTORC1/2, XL388, AKT, autophagy and chemo-sensitization

## Abstract

In the present study, we investigated the activity of XL388, a novel mammalian target of rapamycin (mTOR) complex 1/2 (mTORC1/2) dual inhibitor, in preclinical osteosarcoma (OS) models. XL388 was cytotoxic, cytostatic and pro-apoptotic to multiple established OS cell lines and primary human OS cells. XL388 blocked mTORC1/2 activation and downregulated cyclin D1/B1 expressions in OS cells, leaving AKT Thr-308 phosphorylation un-affected. Intriguingly, AKT1 T308A mutation potentiated XL388-induced cytotoxicity in OS cells. XL388 activated cytoprotective autophagy in OS cells. Autophagy inhibition, either pharmacologically or genetically, augmented XL388-induced anti-OS activity. Further, XL388 oral administration inhibited U2OS xenografts growth in severe combined immuno-deficient (SCID) mice. Such activity was enhanced with co-administration of the autophagy inhibitor 3-methyladenine (3-MA). Similarly, Beclin-1-silenced U2OS xenografts were remarkably more sensitive to XL388. Thus, concurrent blockage of mTORC1/2 with XL388 may have therapeutic value for OS.

## INTRODUCTION

The metastatic or advanced osteosarcoma (OS) is among the most intrinsically resistant malignancies to almost all conventional chemotherapeutic drugs [1, 2]. Therefore, our group [3, 4] and others have been focusing on exploring novel and more efficient anti-OS agents [1]. Molecularly-targeted agents are being tested [1, 2]. Overactivation of mammalian target of rapamycin (mTOR) is observed in many human OS tissues [3, 5–7], which is often associated with cancer progression and poor prognosis [3, 7]. Our group [3] and others have demonstrated that mTOR is vital for several key OS cell functions, such as cell growth, proliferation, and survival, as well as apoptosis-resistance and metastasis.

Preclinical studies have implied a therapeutic value of the mTOR inhibitors for OS [3, 8, 9]. It is known that mTOR forms at least two multi-protein complexes, including the traditional mTOR complex 1 (mTORC1) and later discovered mTORC2 [10–12]. mTOR1, or the rapamycin-sensitive mTOR complex, is assembled with mTOR, Raptor, PRAS40 and possible others [10–12]. On the other hand, mTORC2 is composed of mTOR, Rictor, Sin1 and Protor [10–12]. Both complexes are important for cancer progression, and regulating different and sometime over-lapping cancerous behaviors [10–12].

The clinical use of traditional mTORC1 inhibitors, including rapamycin and its analogs (“rapalogs”), has been limited due to several drawbacks, including the incomplete inhibition of mTORC1, and ineffectiveness to mTORC2 [13]. These led to the development of mTORC1/2 dual inhibitors, also known as the second generation of mTOR inhibitors [13]. Recent studies have characterized XL388 as a selective, highly-potent, and orally available small-molecule ATP-competitive inhibitor of mTORC1/2 [14]. It blocked mTORC1 and mTORC2 activation simultaneously [14]. The IC-50 of XL388 for mTOR is around 10–100 nM [14]. This mTORC1/2 dual inhibitor has displayed anti-tumor activity in preclinical cancer models [14]. In the current study, we explored its activity in preclinical OS models.

## RESULTS

### XL388 is cytotoxic to OS cells

**Figure 1:**
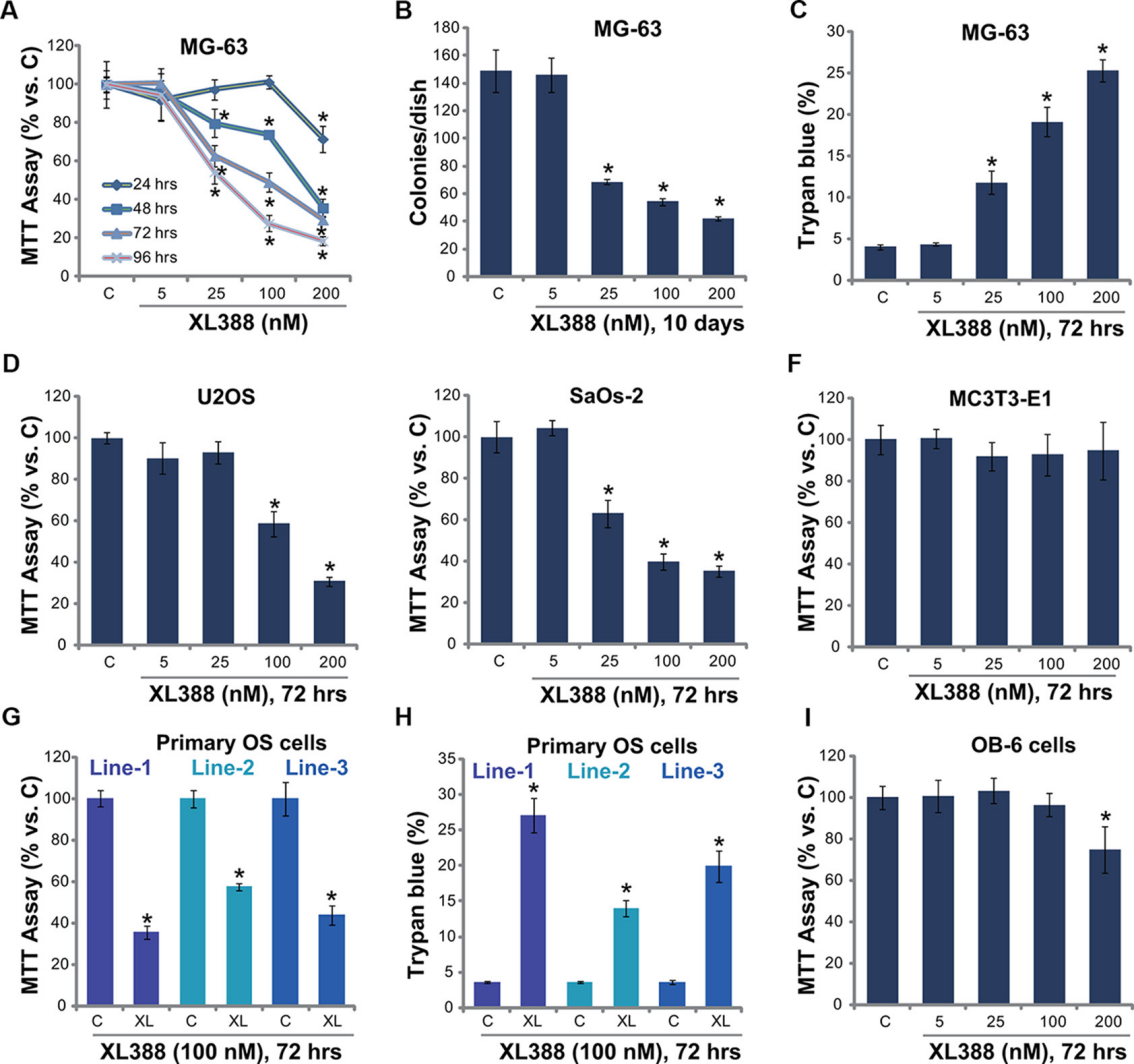
XL388 inhibits OS cell survival-MG-63 (**A–C**), U2OS (**D**) and SaOs-2 (**E**) osteosarcoma (OS) cell lines, the primary human OS cells (line-1/−2/−3) (**G**, **H**), the murine osteoblastic MC3T3-E1 cells (**F**) or the human OB-6 osteoblastic cells (**I**) were treated with applied concentration of XL388, cells were further cultured, and cell survival was tested by MTT assay (A, D–F, G and I) or clonogenicity assay (B); Cell death was tested by trypan blue staining assay (C and H). The data in this and all following figures were representatives of three different experiments. *n* = 5 for each assay. The values were expressed as the means ± SD (Same for all figures). “C” stands for untreated control group (Same for all figures). “XL” stands for XL388 (Same for all figures). “hrs” stands for hours (Same for all figures). *p < 0.05 vs. group “C”.

mTOR is often over-activated in OS [7], and XL388 is novel and selective mTORC1/2 dual inhibitor [14]. The structure and molecular weight of XL388 were presented in Supplementary Figure 1. MTT assay results in Figure 1A demonstrated that XL388 dose-dependently inhibited MG-63 cell survival. At the meantime, a time dependent effect by XL388 was noticed (Figure 1A). Significant MG-63 cytotoxicity was noticed 48-96 hours following XL388 (25–200 nM) treatment (Figure 1A). XL388-induced cytotoxicity against MG-63 cells was also confirmed by the colony formation assay (Figure 1B) and trypan blue staining assay (Figure 1C). With XL388 treatment, the number of viable MG-63 colonies was significantly decreased, yet the trypan blue positively MG-63 cells was increased (Figure 1B and 1C). The similar cytotoxic effect by the novel mTOR inhibitor was also noticed in two other human OS cell lines: U2OS (Figure 1D) and SaOs-2 (Figure 1E). Interestingly, same XL388 treatment showed negligible effect on osteoblastic MC3T3-E1 cells (Figure 1F). One possible reason could be the basal mTOR activation level was very low in MC3T3-E1 cells (Data not shown). Importantly, XL388 was more potent than same concentration (100 nM) of known mTORC1 inhibitors (rapamycin and RAD001) in inhibiting MG-63 and SaOs-2 cell survival (Supplementary Figure 2).

Next, the effect of XL388 on primary human OS cells was also tested. Using the method described, we successfully cultured three lines of primary human OS cells from affected patients (See methods). MTT assay results showed clearly that treatment with XL388 (100 nM) inhibited survival of these primary human OS cells (Figure 1G). Meanwhile, the number of trypan blue positive (“dead”) primary OS cells was increased (Figure 1H). On the other hand, XL388 only induced minor effect on the survival of human OB-6 osteoblastic cells (Figure 1I) and primary murine osteoblasts (Supplementary Figure 2), which showed low basal mTORC1/2 activation (See following). These *in vitro* results indicate that XL388 induces selective and potent cytotoxic effects to cultured human OS cells.

### Caspase-dependent apoptosis activation in XL388-treated OS cells

**Figure 2:**
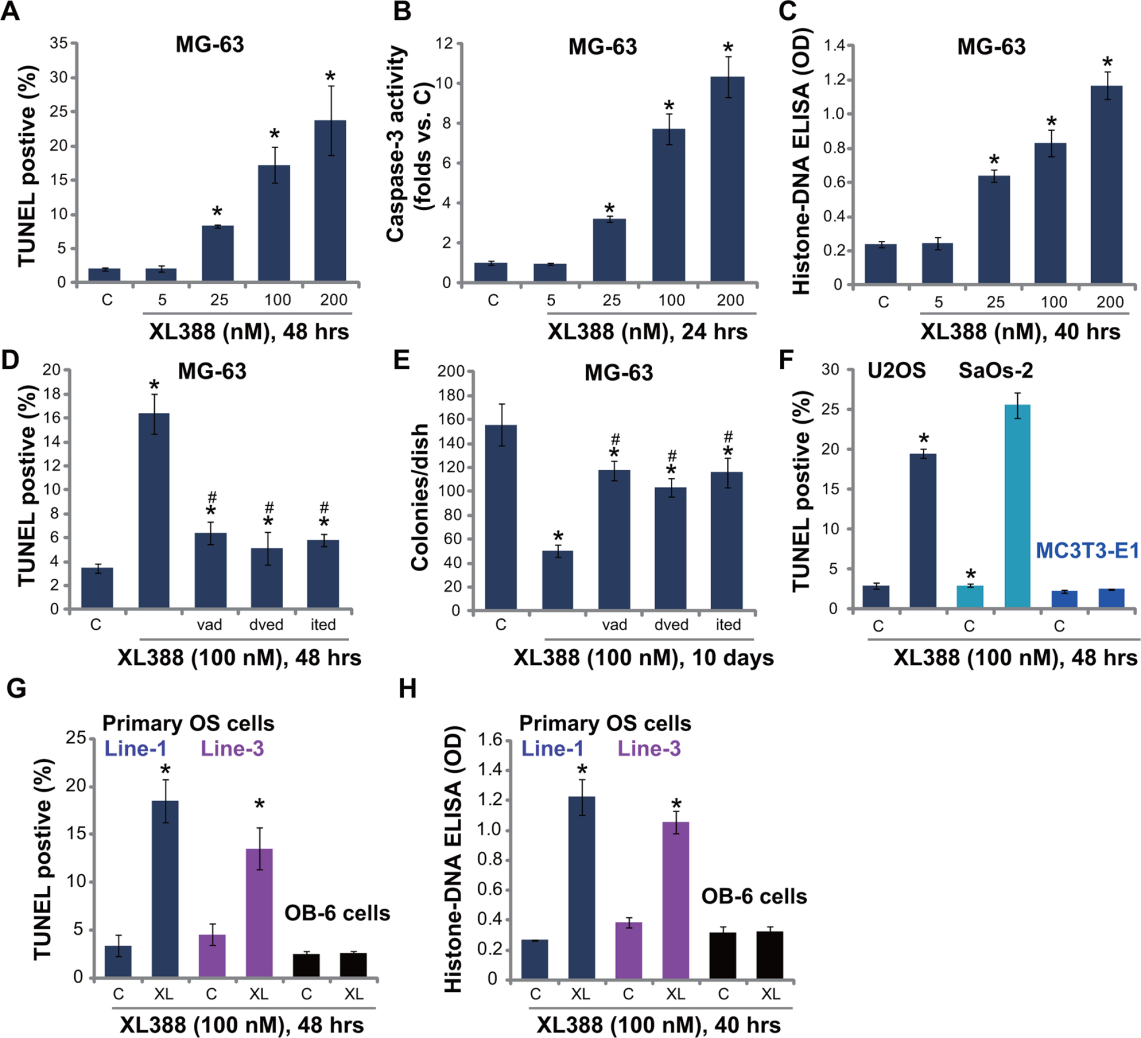
XL388 induces caspase-dependent apoptosis activation in OS cells-MG-63 (**A–C**), U2OS (**F**) and SaOs-2 (F) OS cell lines, the primary human OS cells (line-1/−3) (**G** and **H**), as well as mouse osteoblastic MC3T3-E1 cells (F) and human OB-6 osteoblastic cells (G and H) were treated with applied concentration of XL388, cells were further cultured, and cell apoptosis was tested by listed assays. MG-63 cells, pretreated with z-VAD-fmk (vad, 25 μM), z-DVED-fmk (dved, 25 μM) or z-ITED-fmk (ited, 25 μM) for 1 hour, were stimulated with XL388 (100 nM), TUNEL staining assay (**D**) and colony formation assay (**E**) were performed. *n* = 5 for each assay. *p < 0.05 vs. group “C”. ^#^ *p* < 0.05 vs. XL388 only group (D–E).

XL388-induced inhibition on OS cell survival could be due to apoptosis. We studied the potential effect of XL388 on OS cell apoptosis. As described in our previous study [3], three independent apoptosis assays, including TUNEL staining assay, caspase-3 activity assay and histone DNA ELISA assay, were applied. Results from these assays demonstrated that XL388 induced significant apoptosis activation in MG-63 cells (Figure 2A–2C). XL388 showed a dose-dependent effect in promoting MG-63 cell apoptosis (Figure 2A–2C). To study the effect of apoptosis in XL388-induced cytotoxicity, various caspase-dependent apoptosis inhibitors were utilized [3]. As demonstrated, the pan-caspase inhibitor (z-VAD-fmk, vad), the caspase-3 specific inhibitor (z-DVED-fmk, dved) and the caspase-8 specific inhibitor (z-ITED-fmk, ited) all remarkably inhibited XL388-induced MG-63 cell apoptosis (TUNEL assay, Figure 2D). Importantly, XL388-induced MG63 cytotoxicity (colony number reduction) was significantly alleviated with co-treatment with these caspase inhibitors (Figure 2E). TUNEL staining assay results in Figure 2F demonstrated that XL388 (100 nM) induced apoptosis in other two OS cell lines (U2OS and SaOs-2), but not in non-cancerous MC3T3-E1 cells. Further studies demonstrated the pro-apoptosis activity by XL388 in primary human OS cells (Figure 2G and 2H, Line-2 data not shown). Yet, no significant apoptosis was observed in OB-6 osteoblastic cells after same XL388 treatment (Figure 2G and 2H). Collectively, we show that XL388 induces caspase-dependent apoptosis in cultured OS cells.

### XL388 inactivates mTORC1/2 in OS cells

**Figure 3:**
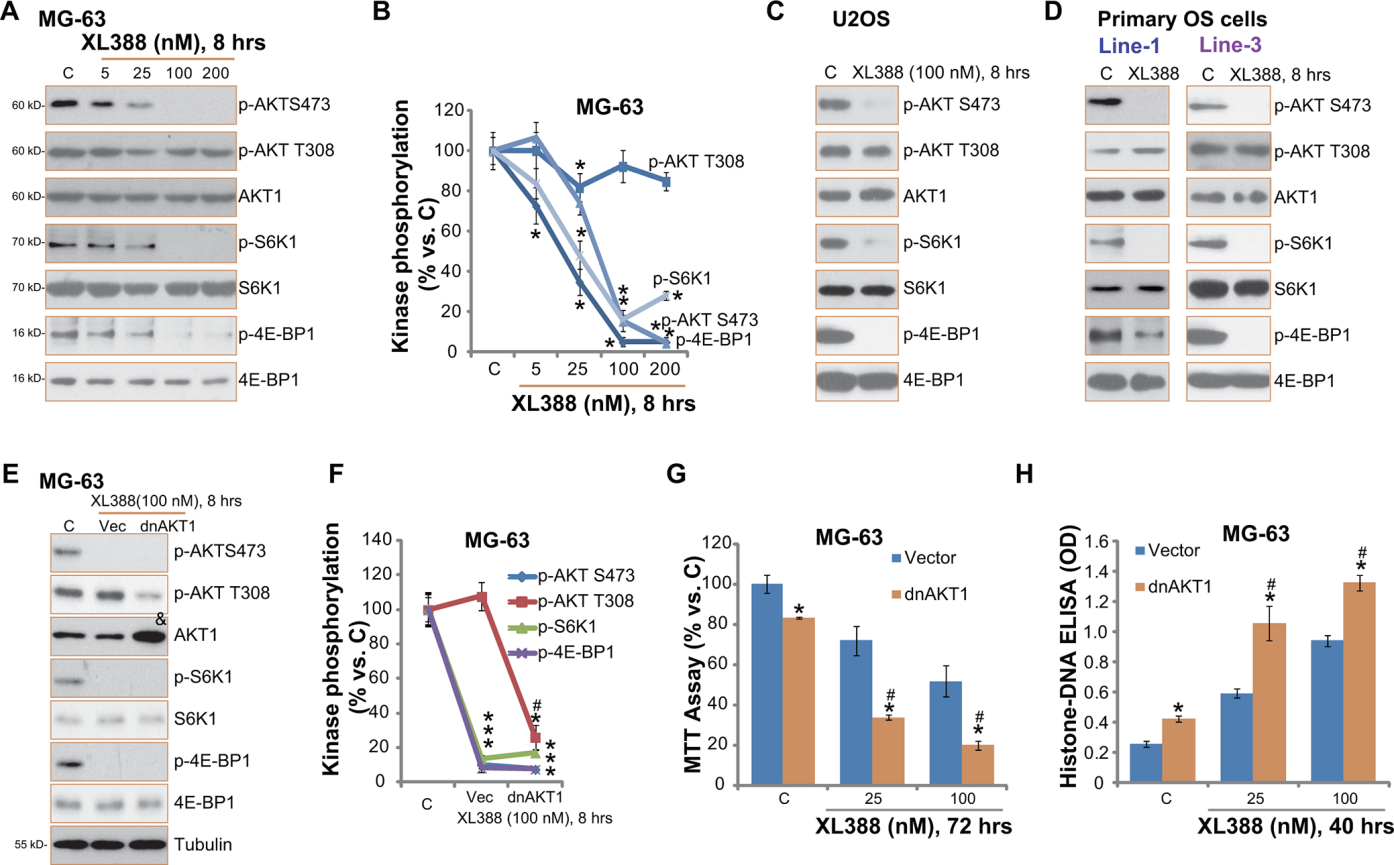
XL388 inactivates mTORC1/2 in OS cells Listed OS cells were treated with applied concentration of XL388 for 8 hours, expressions of listed kinases (*p*- and regular) were tested by Western blots (**A, C** and **D**). Kinase phosphorylation was quantified (**B**). Stable MG-63 cells expressing T308A dominant negative AKT1 (“dnAKT1”) or empty vector (“Vec”, pcDNA3-puro) were treated with or without XL388, cells were further cultured, expressions of listed kinases were tested by Western blots (**E**), kinase phosphorylation was quantified (**F**); Cell survival and apoptosis were tested by MTT assay (**G**) and histone DNA ELISA assay (**H**), respectively. *n* = 3 for each assay. *p < 0.05 vs. group “C”. *^#^p* < 0.05 vs. “Vec” group (**F**–**H**).

XL388 is a mTORC1/2 dual inhibitor [14]. Therefore, we studied the mTORC1/2 activation in XL388-treated OS cells. Phosphorylated- (p-) S6K1 (Thr-389) and p-4E-BP1 (Ser 65) were tested as indicators of mTORC1 activation, and p-AKT (Ser-473) was tested to reflect mTORC2 activity [11]. As shown in Figure 3A, XL388 potently inhibited activation of both mTORC1 and mTORC2 in MG-63 cells. The effect of XL388 on mTORC1/2 activation was again dose-dependent (Figure 3B). Further, mTORC1/2 activation was almost blocked in XL388 (100 nM)-treated U2OS cells (Figure 3C), SaOs-2 cells (Data not shown) and primary human OS cells (Figure 3D). Note that mTORC1/2 activation level in OS cells was significantly higher than that in OB-6 cells and primary murine osteoblasts (Supplementary Figure 3).

On the other hand, AKT Thr-308 phosphorylation was not affected by same XL388 treatment (Figure 3A–3D). Considering that Thr-308 phosphorylation was also critical for AKT activation and OS cell survival [3, 20], we then introduced a dominant negative T308A mutated AKT1 (“dnAKT1”) into MG-63 cells. Western blot results in Figure 3E confirmed dnAKT1 expression (“&” labeled) in the stable MG-63 cells (See methods). Consequently, AKT Thr-308 phosphorylation was dramatically inhibited in dnAKT1-expressing MG-63 cells (Figure 3E and 3F). Significantly, XL388-induced cytotoxicity, evidenced by viability reduction (Figure 3G) and apoptosis ELISA OD increase (Figure 3H), was significantly more potent in dnAKT1-expressing MG-63 cells. Same dnAKT1 experiments were also performed in U2OS cells, and similar results were obtained (Data not shown). Collectively, these results indicate that XL388 inactivates mTORC1/2 in cultured OS cells, and inhibition of AKT Thr-308 phosphorylation could further potentiate XL388′s activity against OS cells.

### XL388 inhibits OS cell cycle progression

**Figure 4:**
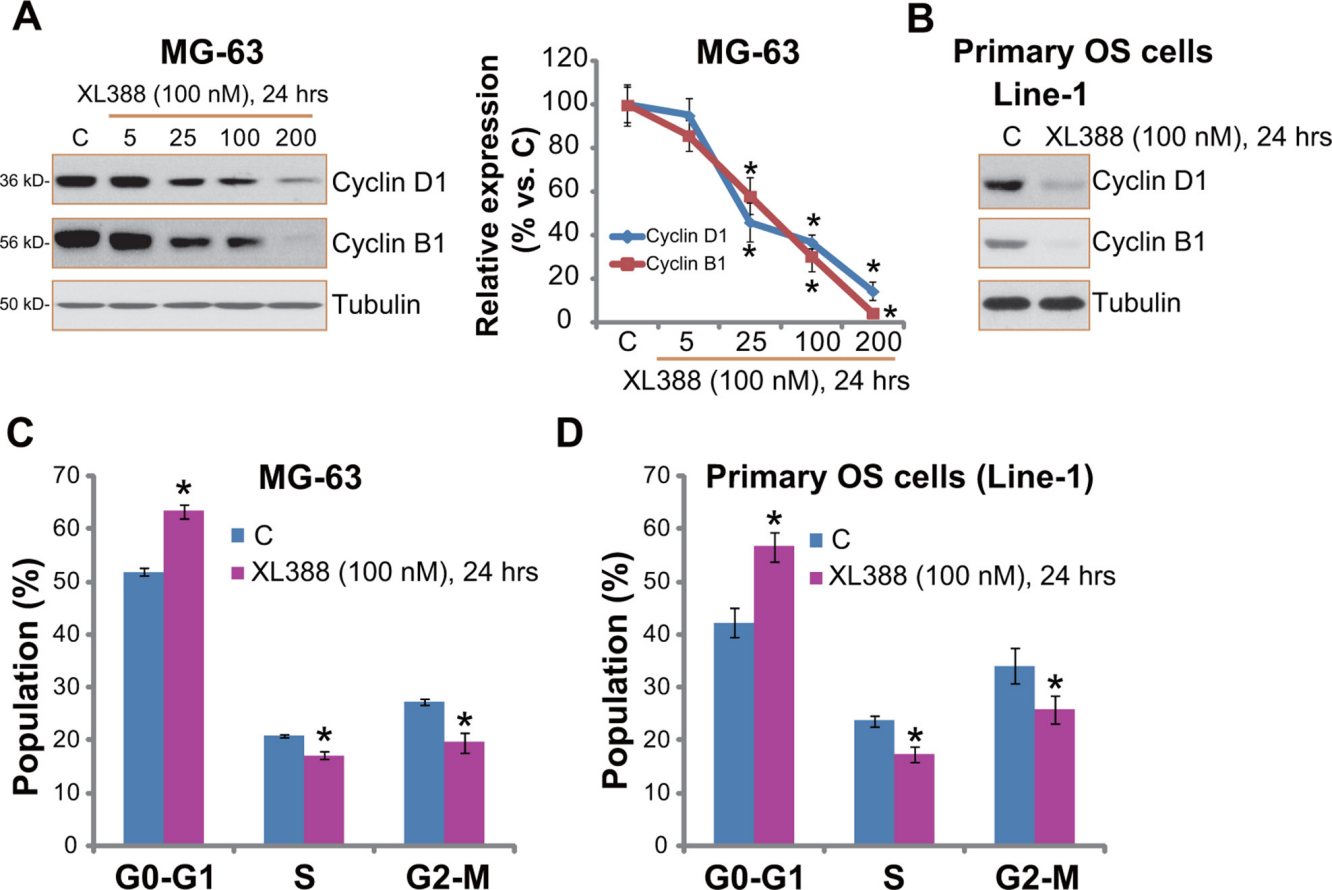
XL388 inhibits OS cell cycle progression Listed OS cells were treated with applied concentration of XL388 for 24 hours, cyclin B1/D1 expression was tested by Western blots (**A** and **B**); Quantitative cell cycle distribution was examined by FACS assay (**C** and **D**). *n* = 3 for each assay. *p < 0.05 vs. group “C”.

mTOR activation is important for expression of several cyclins and cell cycle progression [21]. Our previous study showed that NVP-BEZ235, a novel PI3K/mTOR dual inhibitor, downregulated cyclins expression in cultured OS cells [3]. Here, we showed that cyclin D1 and cyclin B1 expression level was also decreased in XL388-treated MG-63 cells (Figure 4A). Quantified results demonstrated a dose-dependent response by XL388 (Figure 4A, right panel). Similar results were also obtained in U2OS cells (Data not shown) and in primary human OS cells (Figure 4B).

The effect of XL388 on OS cell cycle progression was also analyzed. As demonstrated in Figure 4C, the cell cycle distribution in XL388-treated MG-63 cells was different from that of untreated control cells. The percentage of G1 phase was increased following XL388 treatment, while the S phase and G2-M phase were both decreased (Figure 4C), indicating G1-S arrest. Similar cell cycle results were observed in XL388-treated primary OS cells (Figure 4D). For analyzing cell cycle progression, OS cells were treated with XL388 for 24 hours, when no significant cytotoxicity was noticed (See Figure 1). Together, these results demonstrate that XL388 inhibits OS cell cycle progression.

### XL388 activates autophagy in OS cells

**Figure 5:**
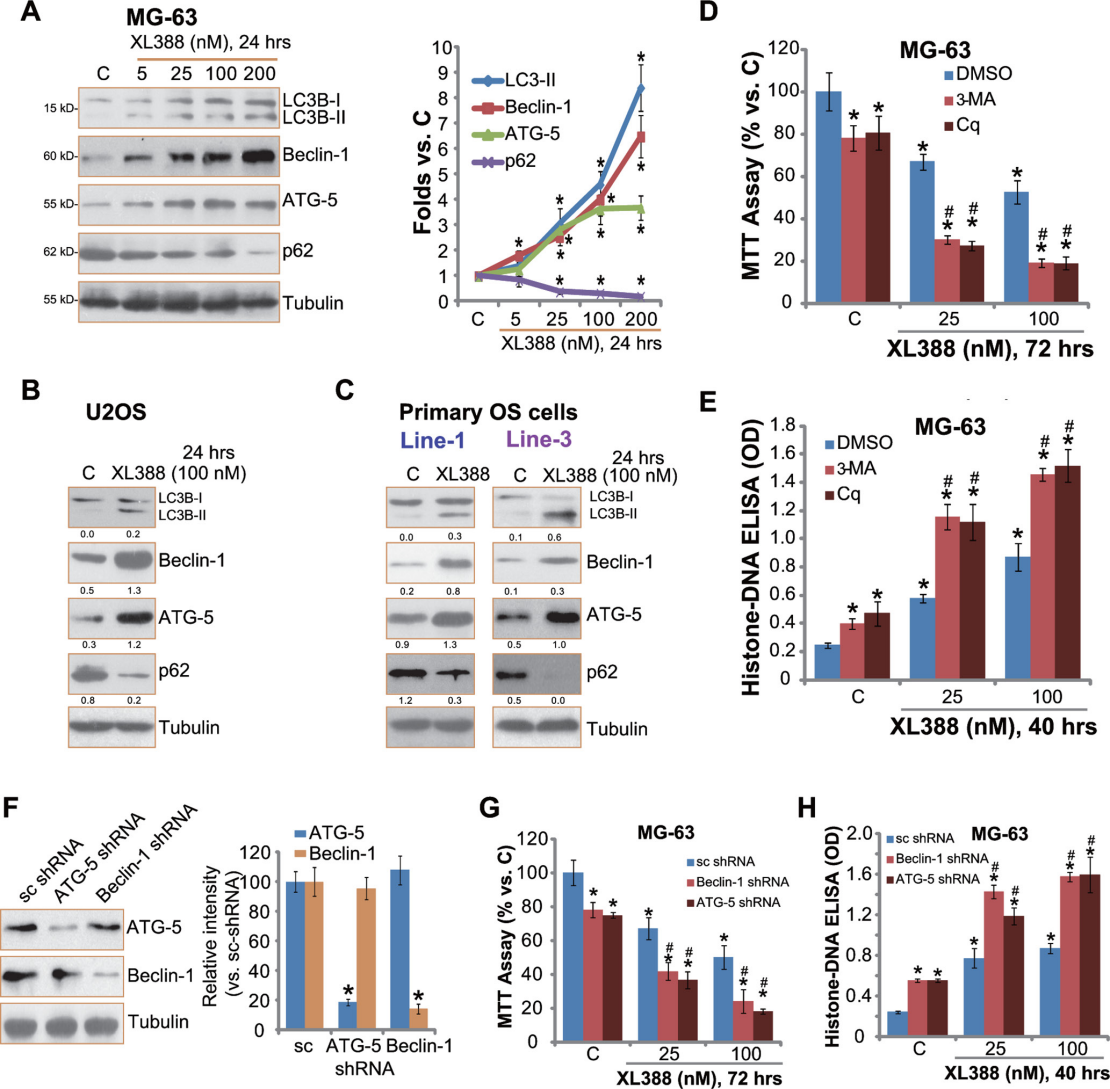
XL388 activates autophagy in OS cells Listed OS cells were treated with applied concentration of XL388 for 24 hours, expression of listed proteins was tested by Western blots (**A**–**C**), quantification was also performed (A–C). MG-63 cells, pre-treated with 3-methyladenine (3-MA, 0.5 mM), chloroquine (Cq, 10 μM), or 0.1% of DMSO (“DMSO”) for 1 hour, were treated with XL388 (25/100 nM) for applied time, cell survival and apoptosis were tested (**D** and **E**). Stable MG-63 cells, expressing scramble shRNA (“sc shRNA”), Beclin-1 shRNA or ATG-5 shRNA (see Beclin-1/ATG-5 expression in (**F**), were treated with or without XL388 (25/100 nM) for applied time, cell survival and apoptosis were tested (**G** and **H**). *n* = 3 for each assay. *p < 0.05 vs. group “C”. ^#^*p* < 0.05 vs. “DMSO” group (**D**–**E**). ^**#**^*p* < 0.05 vs. “sc shRNA” group (G–H).

Existing evidences have shown that a number of mTOR inhibitors could activate autophagy in various cancer cells, which serves as a pro-survival factor counteracting cancer cell death [18, 22, 23]. Next, we tested the potential effect of XL388 on autophagy activation in cultured OS cells. As shown in Figure 5A, expression of autophagy markers, including light chain 3B-II (LC3B-II), Beclin-1 and ATG-5 [24, 25], was significantly increased following XL388 treatment in MG-63 cells (see quantification on the right). Reversely, p62 expression was downregulated (Figure 5A), further suggesting autophagy activation [26]. Similar blot results were also observed in XL388-treated U2OS cells (Figure 5B) and primary OS cells (Figure 5C). These data indicate autophagy activation following XL388 treatment in cultured OS cells.

To study the role of autophagy in XL388-mediated anti-OS activity, pharmacological strategy was applied. Two well-known autophagy inhibitors, including chloroquine (Cq) and 3-methyladenine (3-MA), were applied. Results showed that the co-treatment with the autophagy inhibitors dramatically facilitated XL388-induced survival inhibition (Figure 5D) and apoptosis (Figure 5E) in MG-63 cells, suggesting that autophagy mainly exerted a cytoprotective role, counteracting XL388′s actions in OS cells. Note that 3-MA or Cq alone induced minor cytotoxicity in MG-63 cells, indicating that basal autophagy activation was also important for MG-63 cell survival (Figure 5D and 5E). Similar XL388-sensitizaiton activity by the two inhibitors was also observed in U2OS cells and primary human OS cells (Data not shown).

To exclude the possible off-target effects by the autophagy inhibitors, shRNA method was applied to selectively knockdown autophagy proteins, Beclin-1 and ATG-5, in MG-63 cells. Western blot results in Figure 5F confirmed Beclin-1/ATG-5 downregulation following corresponding shRNA treatment. As a result, XL388-induced MG-63 cytotoxicity (Figure 5G) and apoptosis (Figure 5H) were significantly enhanced with Beclin-1/ATG-5 shRNA silence. Same experiments were also performed in U2OS cells, and similar results were obtained (Data not shown). These results further support the role of autophagy in XL388-mediated activities in OS cells. Together, these results show that XL388 activates cytoprotective autophagy in OS cells, counteracting following cell apoptosis.

### XL388 inhibits U2OS xenografts growth *in vivo*, its activity is further enhanced with autophagy inhibition

**Figure 6:**
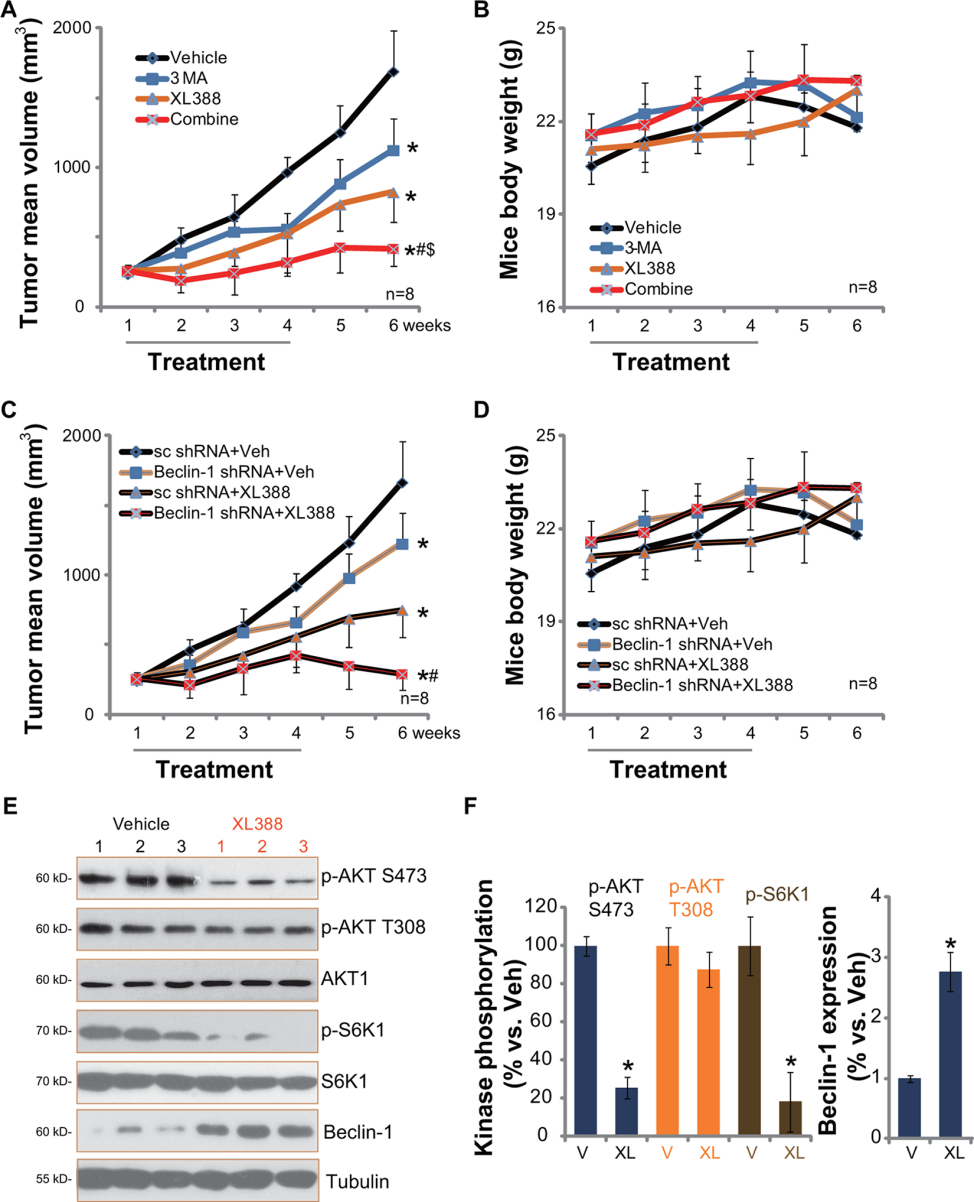
XL388 inhibits U2OS xenografts growth *in vivo*, its activity is further enhanced with autophagy inhibition The U2OS bearing SCID mice (11 mice per group) were administrated with vehicle (“Veh”, saline), XL388 (20 mg/kg, oral gavage, every three days, × 7 times), or plus 3-MA (20 mg/kg, *i.p.* every three days, × 7 times), tumor size was measured every week for a total of 6 weeks (**A**). Mice body weight was also recorded (**B**). Two weeks after initial drug administration, xenografted tumors of three mice per group were isolated and subjected to Western blot assay (**E**, and quantified in **F**) of listed proteins. Stable U2OS cells expressing scramble control shRNA or Beclin-1 shRNA were injected into SCID mice to establish xenograft model, mice were then treated once daily with vehicle (saline) or XL388 (20 mg/kg, oral gavage, every three days, × 7 times), tumor size (**C**) and mice body weights (**D**) were presented. *p < 0.05 vs. “Veh” or “V” group (A and F). **#***p* < 0.05 vs. XL388 only group (A). ^$^*p* < 0.05 vs. 3-MA only group (A). *p < 0.05 vs. “Veh” of “sc shRNA” group (C). **^#^***p* < 0.05 vs. XL388 of “sc shRNA” group (C).

At last, we tested the *in vivo* anti-OS activity by XL388 using the U2OS xenograft SCID mice model [3]. U2OS tumor growth curve results in Figure 6A demonstrated that oral administration of XL388 (20 mg/kg body weight, every three days, × 7 times) significantly inhibited U2OS xenograft growth in SCID mice. Importantly, its activity was further enhanced when combined with the autophagy inhibitor 3-MA (Figure 6A). The combined efficiency was more potent than either single treatment (Figure 6A). 3-MA as a single agent also slightly inhibited U2OS xenograft growth at applied concentration (20 mg/kg, *i.p.*, every three days, × 7 times, Figure 6A) [19]. Note that the mice body weight was apparently not affected by the single or the combined treatment (Figure 6B).

These results indicate that autophagy inhibition could sensitize XL388-induced anti-OS activity *in vivo*. To further support this conclusion, we utilized Beclin-1-shRNA expressing stable U2OS cells to establish xenograft model. Results in Figure 6C showed that Beclin-1-silenced U2OS xenografts grew slower than sc shRNA-expressing U2OS xenografts. More importantly, these Beclin-1-silenced U2OS xenografts were remarkably more sensitive to XL388 (Figure 6C). Once again, mice body weight was not significantly affected by the regimens (Figure 6D).

Next, we studied whether *in vitro* signaling change by XL388 was also seen *in vivo*. Western blots were applied to analyze above signalings in the U2OS xenografts. Results in Figure 6E and 6F demonstrated clearly that XL388 oral administration dramatically inhibited p-S6K1 (mTORC1 activation marker) and p-AKT Ser-473 (mTORC2 activation marker) in U2OS xenografts. p-AKT Thr-308 was again not affected (Figure 6E and 6F). On the other hand, Beclin-1 expression, an indicator of autophagy activation [27], was increased (Figure 6E and 6F). These results indicate that XL388 administration inhibits mTORC1/2 activation, and possibly activates autophagy in U2OS xenografts.

## DISCUSSIONS

Studies have shown that mTOR overactivation is an important contributor of OS progression [7]. There are several limitations when using the traditional mTORC1 inhibitors (rapalogs). First, mTORC1 inhibition by rapamycin or its analogs could lead to feedback activation of PI3K-AKT and several other pro-survival/anti-apoptosis pathways [28, 29]. Second, instead of acting directly on mTOR, rapalogs bind to the cytosolic protein FK-binding protein 12 (FKBP12), leading to partial inhibition of mTORC1 [28, 29]. Third, the mTORC1 inhibitors are often with poor water solubility, and clinical usage is limited [28, 29]. Here we showed that XL388 was significantly more potent than known mTORC1 inhibitors (rapamycin and RAD001) in inhibiting OS cells. Intriguingly, XL388 only exerted minor cytotoxicity to OB-6 osteoblastic cells and primary murine osteoblasts. One possibility is that these osteoblasts showed low basal mTORC1/2 activation (Supplementary Figure 3). Another possibility is that other signalings besides AKT-mTOR are more important to the survival of osteoblasts, which are obviously not affected by XL388.

Recent studies have shown that mTOR inhibition could lead to feedback activation of cytoprotective autophagy, which counteracts the anti-cancer activity by a number of mTOR inhibitors [18]. Here, we showed that XL388 blocked both mTORC1 (p-S6K1/4E-BP1) and mTORC2 (p-AKT-Ser 473) activation in OS cells, which might explain the subsequent autophagy activation. Note that autophagy activation was detected by LC3B-I to LC3B-II conversion, Beclin-1/ATG-5 upregulation, and p62 degradation [24]. XL388-induced autophagy induction was observed not only in established OS cell lines, but also in primary human OS cells.

One important finding of this study is that autophagy inhibition could significantly sensitize XL388-induced anti-OS activity *in vitro* and *in vivo*. Our results are consistent with recent findings showing that autophagy inhibition in combination with mTOR blockage could lead to a profound anti-cancer activity [18, 30, 31]. The autophagy inhibitor chloroquine (Cq) increases intra-lysosomal pH, thus inhibiting autophagic protein degradation [32]. 3-methyladenine (3-MA) is shown to interfere LC3B-I to LC3B-II conversion, thus interposing autophagosome initiation [33]. Both autophagy inhibitors facilitated XL388-induced cytotoxicity in cultured OS cells. More importantly, inhibition of autophagy *in vivo* by oral administration of 3-MA [18] dramatically sensitized XL388-induced activity on U2OS xenograft growth in SCID mice. Further, shRNA-mediated knockdown of Beclin-1/ATG-5 dramatically sensitized XL388′s actions *in vitro*. More importantly, XL388-induced activity *in vivo* was remarkably more potent in U2OS xenografts with Beclin-1 shRNA depletion. Thus, autophagy activation could be a major resistance factor of XL388 in OS cells.

## MATERIALS AND METHODS

### Ethics

All methods listed in the study were carried out in accordance with the approved guidelines by authors' institutions (Medical College of Southeast University and the Second Affiliated Hospital of Soochow University).

### Reagents and chemicals

XL388 was provided by MedChem Express China (Shanghai, China). mTORC1 inhibitors rapamycin and RAD001 were purchased from Selleck (Shanghai, China). The broad caspase inhibitor z-VAD-fmk, the caspase-3 specific inhibitor z-DVED-fmk, and the caspase-8 specific inhibitor z-ITED-fmk were all from Calbiochem (Darmstadt, Germany). Autophagy inhibitors chloroquine (Cq) and 3-methyladenine (3-MA) were obtained from Sigma-Aldrich Co. (St. Louis, MO). Antibodies utilized in this study were all provided by Cell Signaling Technologies (Beverly, MA) [3].

### Cell culture

U2OS, SaOs-2 and MG-63 OS cell lines as well as the murine calvaria-derived osteoblastic MC3T3-E1 cells were maintained and cultured as reported [3, 4]. The OB-6 human osteoblastic cells were purchased from the Cell Bank of Shanghai Institute of Biological Science (Shanghai, China), and were cultured as described [15]. For primary culture of murine osteoblasts, the trimmed calvariae of neonatal mice were digested with 0.1% collagenase I (Sigma) and 0.25% dispase (Sigma). The resolving cell suspensions were neutralized with complete culture medium and were filtered. The calvarial osteoblasts were then resuspended in 10 mL α-MEM containing 15% FBS, and were cultured.

### Culture of primary human OS cells

Surgery-separated human OS tissues were washed, and digested in collagenase I (Sigma)-containing DMEM medium. After 3–5 digestions, the resolving cell suspensions were cultured in complete DMEM medium, containing 10% FBS, 2 mM glutamine, 15 mM HEPES buffer, 0.5 μg/mL hydrocortisone and 2.5 μg/mL insulin, 1.0 μg/mL EGF (Sigma). Passage 3–8 were utilized for further experiments. The protocols were approved by the Internal Review Board (IRB) of all authors' institutions, and were conducted according to the principles expressed in the Declaration of Helsinki. The written-informed consent was obtained from each participant. The tissue specimens were collected from three OS patients. Enneking grading of the OS patients were listed: Patient number 1 (Male, 16 years old): G1/T1/M0; Patient number 2 (Male, 20 years old): G1/T0/M0; Patient number 3 (Male, 19 years old): G2/T0/M0. These patients received no chemotherapy or radiation prior to surgery.

### Cell survival assay

As reported [3, 4], cell survival was tested by the MTT assay.

### Trypan blue staining of “dead” cells

After applied treatment, dead cells were stained by trypan blue dye (Sigma), and the percentage (%) of trypan blue positive cells was recorded.

### Clonogenicity assay

Cells (5 × 10^4^/well) were suspended in 1 mL of DMEM with 1% agar (Sigma), 10 % FBS and with indicated XL388 treatment. The cell suspension was then added on top of a pre-solidified 1% agar in a 100 mm culture dish. The drug containing medium was refreshed every 2 days. After 10-day incubation, the number of remaining colonies were stained and manually counted.

### Western blots

Cells were lysed by the lysis buffer described [3, 4]. Samples (20–30 μg) were separated by 8–15% SDS-PAGE gel, and transferred onto polyvinylidene fluoride (PVDF) membranes (Millipore, USA). Afterwards, the membranes were blocked, and incubated overnight at 4°C with the indicated primary antibodies. Secondary antibodies were conjugated to horseradish peroxidase (Santa Cruz). Detection was accomplished by chemiluminescence with ECL (GE Healthcare). Quantification of bands was performed as described [3, 4].

### TUNEL assay of apoptosis

Cell apoptosis was detected by the TUNEL (Terminal Deoxynucleotidyl Transferase dUTP Nick End Labeling) *In Situ* Cell Apoptosis Detection Kit (Roche, Shanghai, China), according to the manufacturer's instructions. The detailed protocol was described in our previous study [3].

### Caspase-3 activity assay

Cytosolic proteins from approximately 2 × 10^6^ cells were extracted [3, 4]. Twenty μg of cytosolic extracts were added to the caspase assay buffer [3, 4] with Ac-DEVD-AFC (15 μg/mL) (Calbiochem) as the substrate. After incubation, the amount of released AFC was measured using a spectrofluorometer (Thermo-Labsystems, Helsinki, Finland).

### Histone/DNA ELISA for detection of apoptosis

The Cell Apoptosis Detection ELISA Kit (Roche) was utilized to quantify cell apoptosis according to the protocol provided by the manufacturer. Detailed procedure can be found in our studies [3, 4].

### Cell cycle analysis

After treatment, cell cycle was tested by propidium iodide (PI) flow cytometry assay. The detailed protocol was described previously [3].

### Stable shRNA knockdown of Beclin-1 and autophagy-related protein (ATG)-5

The lentiviral particles with Beclin-1 shRNA (sc-29797-V) or the scramble control shRNA (“sc shRNA”, sc-108065) were purchased from Santa Cruz Biotech (Santa Cruz, CA). The human ATG-5 shRNA containing lentivirus were designed and provided by Genechem (Shanghai, China), the ATG-5 shRNA sequences were previously reported [16]. The lentiviral shRNA were added to the cultured OS cells for 36 hours, and stable clones expressing sc-, Beclin-1- or ATG-5-shRNA were selected by puromycin (5.0 μg/mL, Sigma). Cells were cultured in puromycin-containing medium for a total of 10–14 days, and the resistant stable colonies were formed. The expression of targeted protein (Beclin-1/ATG-5) in stable cells was always checked by Western blot.

### AKT T308A mutation

pcDNA3-AKT1 T308A was provided by Genechem (Shanghai, China) through the *in vitro* site-directed mutagenesis system. pcDNA3-AKT1 T308A-puro or the empty vector (pcDNA3-puro) was transiently transfected into MG-63 cells. Transfection was carried out in DMEM with 2% FBS (without antibiotics) utilizing Lipofectamine Reagent (Invitrogen). Stable cells were again selected by puromycin (5.0 μg/mL) as described above. AKT1 expression and Thr-308 phosphorylation in stable cells were verified by Western blots.

### Mice U2OS xenograft

As described previously [3], CB.17 severe combined immuno-deficient (SCID) male mice (5–6 weeks old) were maintained at the animal facility of Southeast University of China (Nanjing, China). Mice were injected subcutaneously (*s.c.*) into the right flanks with 3 × 10^6^ U2OS cells. When xenografts were established at about 250 mm^3^ in volume, the SCID mice (11 mice per group) were administrated with XL388 (20 mg/kg in saline, oral gavage) [17], or plus 3-MA (20 mg/kg in saline, intraperitoneal injection) [18, 19], which were both freshly prepared and given once every 3 days for a total of 21 days. Control mice received vehicle (saline) only. The xenografted tumor diameter was measured every 7 days. Tumor volumes (mm^3^) and mice body weights (g) were recorded as described [3, 4]. The animal protocols in the study were in accordance with the Institutional Animal Care and Use Committee (IACUC), and were approved by authors' institutions.

### Statistics

The data presented were mean ± standard deviation (SD). Statistical differences were analyzed by one-way ANOVA followed by multiple comparisons performed with post hoc Bonferroni test (SPSS version 16). The duration of treatment and concentration of agents were chosen based on pre-experiment results. Values of *p* < 0.05 were considered statistically significant.

## CONCLUSIONS

Recent genetic screen studies have implied that AKT-mTOR signaling is vital for OS development and metastasis [14, 34]. Importantly, a recent Phase II clinical trial study has shown that administration of mTOR inhibitor ridaforolimus improved the progression-free survival (PFS) of patients with advanced OS [35]. The present study showed that XL388 potently inhibited AKT (Ser-473) and mTOR activation, and suppressed OS cell proliferation *in vitro* and *in vivo*. Therefore, XL388 could be further tested as a potential treatment for OS.

## SUPPLEMENTARY MATERIALS FIGURES


