# Supplementary material for: The anti-cancer activity of the mTORC1/2 dual inhibitor XL388 in preclinical osteosarcoma models
Source: Oncotarget. 2016 Jul 2;7(31):49527–38. doi: 10.18632/oncotarget.10389 (PMC5226526; doi:10.18632/oncotarget.10389)
Supplement: Supplementary file 1 [file oncotarget-07-49527-s001.pdf]

The anti-cancer activity of the mTORC1/2 dual inhibitor XL388 in preclinical osteosarcoma models

Supplementary Materials

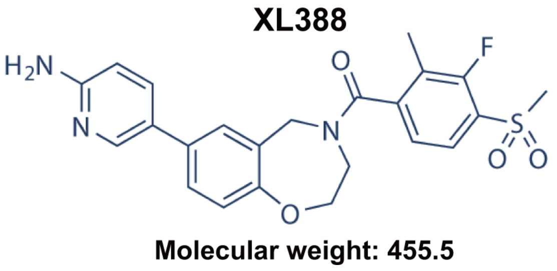

Supplementary Figure S1: The structure and molecular weight of XL388.

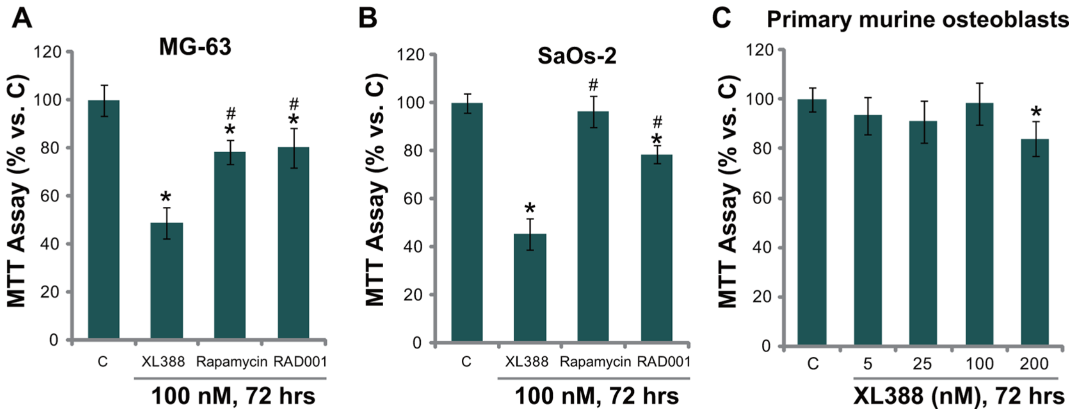

Supplementary Figure S2: MG-63 (**A**), SaOs-2 (**B**) cells or primary murine osteoblasts (**C**) were treated with applied concentration of XL388, rapamycin or RAD001, cells were further cultured for 72 hours, and cell survival was tested by MTT assay. $^{*}p < 0.05$ vs. group "C". $^{\#}p < 0.05$ vs. XL388 group (A and B).

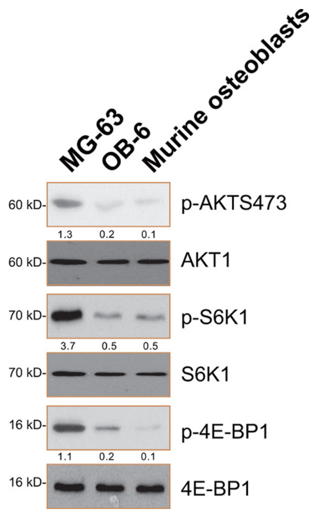

Supplementary Figure S3: Expression of listed proteins in listed cells was tested by Western blots. Kinase phosphorylation was quantified (vs. regular kinase).
